# Biased Sampling and Causal Estimation of Health-Related Information: Laboratory-Based Experimental Research

**DOI:** 10.2196/17502

**Published:** 2020-07-24

**Authors:** María Manuela Moreno-Fernández, Helena Matute

**Affiliations:** 1 Departamento de Fundamentos y Métodos de la Psicología Faculty of Psychology and Education University of Deusto Bilbao Spain

**Keywords:** information sampling, causal illusion, causal bias, health information, health communication

## Abstract

**Background:**

The internet is a relevant source of health-related information. The huge amount of information available on the internet forces users to engage in an active process of information selection. Previous research conducted in the field of experimental psychology showed that information selection itself may promote the development of erroneous beliefs, even if the information collected does not.

**Objective:**

The aim of this study was to assess the relationship between information searching strategy (ie, which cues are used to guide information retrieval) and causal inferences about health while controlling for the effect of additional information features.

**Methods:**

We adapted a standard laboratory task that has previously been used in research on contingency learning to mimic an information searching situation. Participants (N=193) were asked to gather information to determine whether a fictitious drug caused an allergic reaction. They collected individual pieces of evidence in order to support or reject the causal relationship between the two events by inspecting individual cases in which the drug was or was not used or in which the allergic reaction appeared or not. Thus, one group (cause group, n=105) was allowed to sample information based on the potential cause, whereas a second group (effect group, n=88) was allowed to sample information based on the effect. Although participants could select which medical records they wanted to check—cases in which the medicine was used or not (in the cause group) or cases in which the effect appeared or not (in the effect group)—they all received similar evidence that indicated the absence of a causal link between the drug and the reaction. After observing 40 cases, they estimated the drug–allergic reaction causal relationship.

**Results:**

Participants used different strategies for collecting information. In some cases, participants displayed a biased sampling strategy compatible with positive testing, that is, they required a high proportion of evidence in which the drug was administered (in the cause group) or in which the allergic reaction appeared (in the effect group). Biased strategies produced an overrepresentation of certain pieces of evidence at the detriment of the representation of others, which was associated with the accuracy of causal inferences. Thus, how the information was collected (sampling strategy) demonstrated a significant effect on causal inferences (*F*_1,185_=32.53, *P*<.001, η^2^^p^=0.15) suggesting that inferences of the causal relationship between events are related to how the information is gathered.

**Conclusions:**

Mistaken beliefs about health may arise from accurate pieces of information partially because of the way in which information is collected. Patient or person autonomy in gathering health information through the internet, for instance, may contribute to the development of false beliefs from accurate pieces of information because search strategies can be biased.

## Introduction

### Background

In recent years, we have witnessed an increase in diseases, such as measles, that had almost been eradicated in the Western world [1]. One of the factors underlying this outbreak is the antivaccination movement, led by individuals who do not adhere to recommendations for vaccinations (for themselves or their children). Reluctance with respect to vaccination led to the World Health Organization listing vaccine hesitancy as 1 of 10 threats to global health in 2019 [2]. Although vaccine hesitancy is a complex phenomenon [3], common reasons for refusing vaccination are the underestimation of its benefits or the overestimation of its negative side effects; a high proportion of these concerns are based on information disseminated by the media or received from acquaintances [4]. Moreover, previous research [5] has shown that people use the internet as an information source about vaccines, and that side effects and possible negative outcomes of vaccination are one of the most searched topics.

In fact, the internet is an important source of health-related information [6-11]. Newly diagnosed cancer patients perceive the internet as a tool for acquiring information and for making informed decisions [12-14]; patients with diabetes use the internet to seek general information about the disease or about treatment options [15]. The relevant role of the internet, in this context, is not restricted to pathological states. Women who are pregnant use the internet to get informed about topics such as fetal development or to make pregnancy-related decisions [6,16], and after pregnancy, parents use the internet to retrieve health information regarding their infants [8,17].

The internet has accessibility, anonymity, and interactivity as advantages, but these advantages do not come without risks [18]. Some of these risks, such as information overload or lack of credibility, can be considered intrinsic limitations of the internet as a source for health information; however, there are other risks that are based on human skills and cognitive abilities. For example, the huge amount of information available on the internet forces users to engage in an active process of information selection to filter content. As we will discuss later, this information sampling process may play a crucial part in establishing and maintaining mistaken beliefs.

### Evaluating the Risks and Benefits of Medical Treatments

When people judge the risks and benefits of a treatment option, they infer the causal relationship between two events—the treatment and its effect. Unfortunately, causal inferences of this kind are highly difficult because, among other reasons, causality is not directly observable; rather, it must be inferred from cues such as contingency [19,20]. The principle of contingency posits that, unless hidden factors are at play, all causes correlate statistically with their effects.

**Figure 1:**
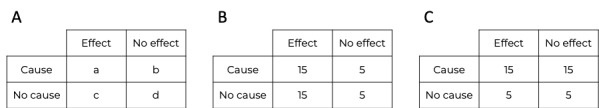
Contingency matrices where (A) shows the four information types as a function of whether the cause and the effect are present, (B) shows an example with a high probability of the effect with null contingency, and (C) shows an example with a high probability of the cause with null contingency.

Consider the simplest case in which a person may try to judge the effect of only one factor (cause) on one given outcome (effect). For the sake of simplicity, it can be assumed that both cause, *C*, and effect, *E*, are binary variables—they either occur or do not occur. In this situation, the person can collect information that fits into one of the following four possibilities (Figure 1, panel A)—*type a*, in which both the cause and the effect occur; *type b*, in which only the cause occurs; *type c*, in which only the effect but not the cause occurs; and *type d*, in which neither the cause nor the effect occurs—which define the cause and effect contingencies. Although different indices have been proposed to represent contingency [21,22], the most popular is, perhaps, the Δ*p* index [23] which is computed as the difference between the probability of the effect conditional on occurrence of the cause, *E*|*C*, and the probability of the effect conditional on absence of the cause, *E*|~*C*, as shown in the equation,

Δ*p* = *p*(*E*|*C*) − *p*(*E*|~*C*) = *a*/(*a* + *b*) – *c*/(*c* + *d*)

where Δ*p* can take on values between –1 and 1. Positive values indicate a generative relationship, and negative values indicate a preventive relationship. When the potential cause and the effect are not related to each other, the index equals zero and contingency is null.

Previous research [23-28] has shown that people are sensitive to contingency between events, and that contingency is used as a cue to make causal inferences; however, under some circumstances, people systematically deviate from the normative standard. Researchers have described two systematic deviations: the influence of the probability of effect occurrence [29-34], when the effect occurs frequently, the causal relationship tends to be overestimated (Figure 1, panel B); and the influence of the probability of occurrence of the cause [24,35,36], when the probability of the cause is high, the contingency perceived between cause and effect is also high (Figure 1, panel C). These biases can be detected even if the contingency between the cause and the effect is null, leading to causal illusions [37]*.*

### Health-Related Information Seeking and Causal Illusions

Imagine someone who is worried about the potential relationship between a vaccine and autism. Usually, it is not possible to evaluate the effects of the vaccine by administering and not administering the vaccine and observing the outcomes since people rarely have the opportunity to conduct a randomized controlled trial. Instead, people will search for information about the relationship between the vaccine and the side effect by consulting an expert, by consulting a friend, or by searching the internet.

Perhaps the most obvious concern about internet-sourced information is the lack of quality control. Internet users may come across and trust information that is not supported by evidence; however, the act of information seeking may entail additional and specific concerns. For example, people who worry about the safety of vaccination and its relationship with autism may look for information about the vaccine (exploring its side effects and the probability of experiencing those side effects, etc). If they are concerned about autism, they may focus their search on autism (exploring which factors have been related with the development of autism or what the proportion of vaccinated children is among those who were diagnosed with autism). In the former, cause (ie, the vaccine) is the cue that guides the search, while in the latter, effect (ie, autism) is the cue that guides the search.

The sampling strategy (how people search for information) will affect their final inference about the relationship between vaccination and autism. If people search for information about the vaccine (ie, the potential cause of autism), they may introduce the name of the vaccine on the search engine, and they will mostly retrieve instances of type a and type b information. This information will allow them to make a general estimation of the probability of the effect (ie, autism) when the potential cause (ie, the vaccine) has been presented; however, in this case, their sampling strategy is biased toward the cause, and therefore, no information about the effect in the absence of the cause, that of either type c or type d, is collected. This will eventually bias their judgements. Indeed, even when sampling is not completely biased toward the cause and some type c or type d instances are collected, it has been repeatedly shown that the higher the tendency to sample information about the cause, the higher the probability of overestimating the cause–effect relationship [37-39].

In our example about the effects of vaccination, this strategy may, nevertheless, be considered as not particularly dangerous. The prevalence of autism spectrum disorders is actually low (1 out of 160 children) [40]; therefore, this sampling strategy will retrieve more type b information than that of type a. In this example, the low base rate of the effect may protect people from developing a causal illusion [41], but in other cases, this protection does not exist (imagine, for example, potential effects such as nausea, high temperature, headache, or any other common effect).

As previously noted, people may also gather information using the effect as their cue for sampling; they may search using terms related to the effect rather than those related to the cause. If people use this sampling strategy for collecting information, they may learn which factors have been associated with autism and will discover that many children among those who developed autism spectrum disorders had been vaccinated. Thus, information sampling will be biased, overrepresenting information in which the effect (ie, autism) is present. In the long run, this strategy will increase the proportion of type a and type c information (relative to that of type b and type d) and will favor a sampling-induced overestimation of the relationship (Figure 1, panel B). This sampling-induced illusion may explain how concerned and educated parents end up overestimating the potential risks of vaccination [42]. Since many countries usually have systematic immunization programs, when information sampling is biased toward effect, the probability of collecting type a information (ie, cases in which autism and vaccination coincide) is even higher than the probability of collecting type c information (ie, cases in which autism occurs in the absence of vaccination). This increases the probability of overestimating the link between vaccination and autism—ie, the probability of experiencing a causal illusion.

The vaccination and autism example illustrates quite well how information sampling may become a crucial element for establishing and maintaining mistaken beliefs; however, the biases in sampling strategies can be extended to a wide range of health issues; people interested in assessing the causal relationship between any common behavior and an infrequent disease will find a high proportion of information where the behavior and the disease coincide if they use the effect as a cue in their internet search. Correspondingly, people using the cause to guide their internet search may end up neglecting the base rate of the effect and end up overestimating the causal relationship when the effect is frequent [43]. For example, a recent study [44] which tracked internet-browsing behavior in a controlled setting showed that when women were required to consult the internet for health information after the hypothetical onset of an unfamiliar breast change (eg, nipple rash), most participants used rash-related search terms (a cue-guided sampling strategy), and the majority accessed websites containing breast cancer information with National Health Service Paget disease of the nipple being the most visited site. In this situation, even when information is accurate, the potential relationship between both events could be overestimated at a substantial emotional cost. Note that, if considering no other information, a nipple rash may be produced by other skin conditions with high incidence rates (such as eczema) rather than by Paget disease, which is a rare type of breast cancer [45].

Information sampling biases may also affect inferences about treatment effectiveness; it is also possible for a biased sampling strategy to induce a perception that underestimates a treatment that is actually effective, or a perception that overestimates the effectiveness of alternative practices proven by clinical trial to perform no better than placebos [39,46].

### Study Goals

As previously described, research on contingency learning has demonstrated how people may use different pieces of information to infer causal relationships [19,20,47]. We have also mentioned that individual behavior may bias information sampling, and consequently, causal inferences; however, these behavior-induced causal illusions have only been explored in situations in which the potential cause was used to guide information sampling. As far as we know, the influence of an equivalent effect-driven sampling has not yet been explored. In addition, these causal illusions have been explored using procedures that usually include motivational components or additional goals which may affect information gathering and causal inferences. For example, participants may be required to evaluate the effects of a fictitious medicine while at the same time trying to heal as many patients as possible [48,49]. It is not clear whether a causal illusion can be detected in an information sampling setting when motivational components or secondary goals are removed, or when sampling strategies are guided by the effect rather than by the cause. The research reported herein explores these two possibilities.

## Methods

### Participants

A sample of adults (N=193) with a mean age of 34.07 (SD 11.41) years consisting of women (83/193, 43.0%), men (109/193, 56.5%), and one (1/193, 0.5%) nonbinary participant were recruited via Prolific Academic internet platform [50]. They were compensated £0.75 (US $0.93) for their participation, which worked out to approximately £5.01 ($6.19) per hour. Enrollment was only offered to individuals in Prolific Academic’s pool whose first language was English (to ensure that instructions were correctly understood) and to individuals who had not taken part in previous studies carried out by our research team. We did not use any exclusion criteria (all participants were included in reporting). Participants were randomly assigned to experimental groups—a cause group (n=105) and an effect group (n=88).

### Instruments

Because of ethical considerations, we avoided using a real-world example in this experiment and instead used a simplified fictitious scenario that is often used in causal learning research; we adapted the allergy task [20,51] for presentation as a web app based on World Wide Web Consortium standards [52]. A demonstration of the program can be downloaded from the Open Science Framework [53].

### Experimental Design and Procedure

We adapted the allergy task [20,51] to make it akin to an information gathering situation with no goal other than that of assessing the causal link between two events. This procedure has been widely used in causal learning research and allows for the assessment of causal illusions while controlling other relevant parameters, which ultimately ensures a high degree of internal validity.

The procedure was set to allow for cause-driven and effect-driven sampling by including a reduced number of changes. Participants were required to gather information to discover whether a fictitious drug (ie, “Batatrim”) caused a fictitious allergic reaction (ie, “Lindsay syndrome”). Participants in the cause group were allowed to sample information based on the potential cause, therefore, could choose to retrieve patient medical records based on patient treatment (whether the patient was treated with Batatrim or not treated with Batatrim), whereas participants in the effect group were allowed to sample information based on the effect, therefore, could choose to retrieve the records based on the development of the syndrome (whether the patient developed Lindsay syndrome while hospitalized or did not). Detailed instructions for the task can be found in Multimedia Appendix 1.

The probability of the cause was under participant control in the cause group whereas the probability of the effect was under participant control in the effect group. Information presented objectively reflected the absence of a causal link between the two events. The probability of the effect in cause group and the probability of the cause in effect group were fixed to 0.75. This design allowed for the evaluation of sampling-dependent causal illusion in a null contingency situation with a high probability of the outcome (cause group) and with a high probability of the cause (effect group).

We expected causal estimations to vary depending on sampling strategy. For participants in the cause group, we expected estimations to increase as the participants increased the probability of the cause—the more biased the participant behavior toward cause-present events, the higher the probability of experiencing causal illusion. Analogously, for participants in the effect group, we expected estimations to increase as participants increased the probability of the effect, also resulting in an increased likelihood of experiencing causal illusion. The procedure used in this study was approved by the ethical review board of the University of Deusto.

### Learning Task

**Figure 2:**
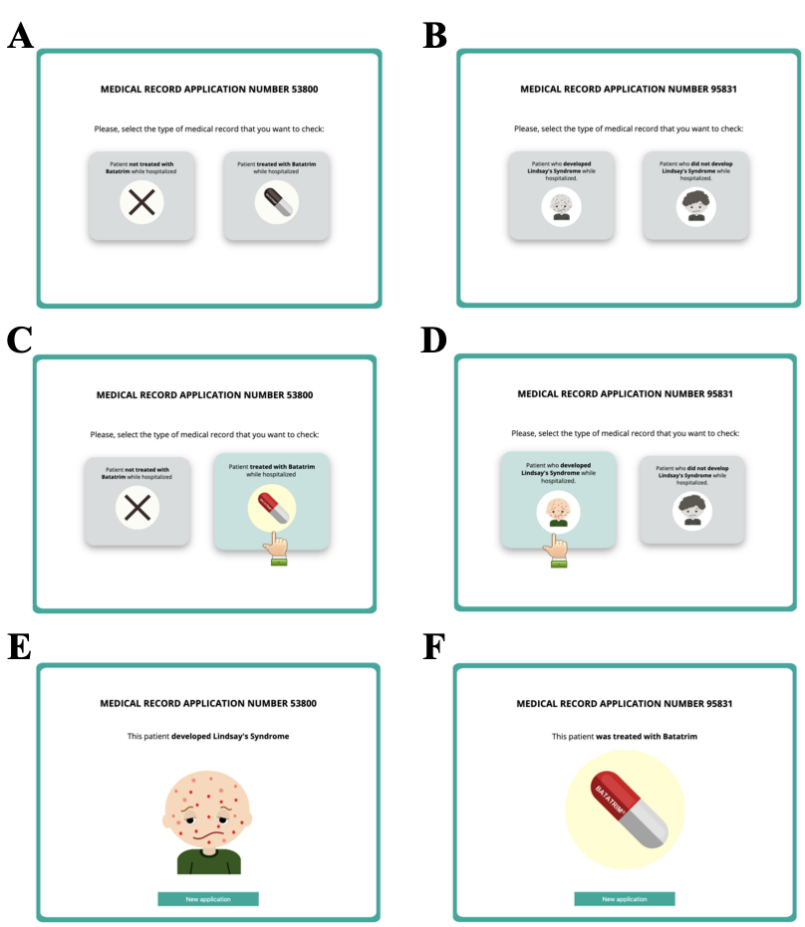
The sequence of events within a trial presented to the cause group (panels on the left) and to the effect group (panels on the right).

A series of 40 patient records was presented, each in a separate trial. Each trial started with a screen on which participants were required to indicate which type of medical record they wanted to check by pressing one of two buttons (Figure 2, panels A and B). The location of each button (left or right) was randomized for each participant. When participants moved the cursor over the buttons, the button colored and zoomed, and a hand pointer appeared to indicate that a response could be made (Figure 2, panels C and D). Once a button was clicked, all the information presented on the screen was removed (with the exception of the sentence stating the medical record application number which remained for esthetic purposes, and for which a random number was used). The information that had been removed was replaced with information about the syndrome when viewed by those in the cause group, or about the treatment when viewed by those in the effect group (Figure 2, panels E and F). One second later, a green button with the words “New application” was presented. After clicking this button, all elements were removed, and after one second, a new trial (a new patient record) appeared. There was no time limit for progressing through the task.

**Figure 3:**
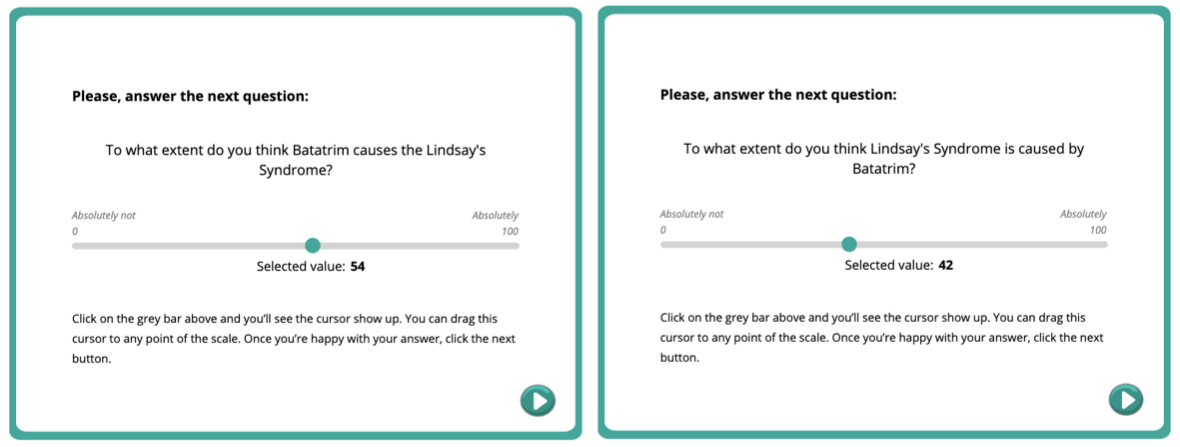
The final screen where the causal relation between Batatrim and Lindsay syndrome is assessed. The question is shown worded as cause-to-effect (left) or effect-to-cause (right).

When participants completed the training stage, they were required to use a 100-point scale to make a global estimation about the causal relationship between Batatrim and Lindsay Syndrome. The question was formulated in the direction of either cause-to-effect “To what extent do you think Batatrim causes the Lindsay syndrome?” or effect-to-cause “To what extent do you think Lindsay syndrome is caused by Batatrim?” The format of the question was randomized among participants (Figure 3). In both cases, the response scale was from 0 (absolutely not) to 100 (absolutely).

### Measures

Causal estimations at the end of the learning task were used as a measure of causal inference [39]. For each participant, we also calculated a sampling strategy index and a measure of experienced contingency.

As previously described, participants in the cause group could choose records based on patient treatment (patients treated or not treated with Batatrim), while participants in the effect group could choose records based on the development of the syndrome (patients who developed or who did not develop the syndrome). Participants could display an unbiased information gathering strategy, asking for a similar proportion of records in both categories; however, it was also possible for participants to display a biased sampling strategy, ie, to preferentially ask for one type of medical record more often than for the other. To measure bias in the information sampling strategy, we calculated a sampling strategy index from training responses as the probability of choosing records of patients treated with Batatrim (in the cause group) or records of patients who developed Lindsay syndrome (in effect group); therefore, the sampling strategy index could range between 0 and 1. Values near 1 indicated a strong preference for checking the medical records of patients treated with Batatrim or patients who developed the syndrome (depending on the group). Values near 0 indicated the opposite strategy, that is, a preference for checking medical records of patients who were not treated with Batatrim or who did not develop the syndrome. A value of 0.5 indicated an unbiased strategy with no preference for either of the two strategies. The higher the index, the higher the probability of retrieving a medical record in which the potential cause and the consequence coincide (type a information), and consequently, the higher the probability of developing a causal illusion.

Additionally, and given that participants could decide which type of medical record they wanted to check, experienced contingency could depart from the programmed value (Δ*p*=0) and also affect their causal estimations [35,54]; therefore, a measure of experienced contingency was calculated (Δ*p* using the actual number of type a, b, c, and d trials to which each participant was exposed).

### Statistical Analysis

Unless noted otherwise, *P*<.05 was deemed as statistically significant. Two-tailed independent *t* tests were used to determine if sampling strategy indices were significantly different from 0.50 (neutral strategy) in either group.

A 2×2 analysis of variance (ANOVA) was performed to assess the effect of group (cause versus effect) and button position (left versus right) on information sampling strategy (sampling strategy index).

An analysis of covariance (ANCOVA) was performed using group (cause or effect) and directionality in which the causal estimation was required (cause-to-effect or effect-to-cause) as fixed factors and information sampling strategy (sampling strategy index) as a covariate to determine the effect on causal estimation. We expected causal estimation to vary as a function of sampling strategy index in both groups—the higher the index, the stronger the causal overestimation of the relationship between the cause and the effect. Additionally, and since the probability of the outcome in the cause group and the probability of the cause in the effect group were fixed at the same high rate, *p*(*C*)=*p*(*E*)=0.75, we explored whether the effect of sampling strategy on causal estimations was equivalent in both groups. We also explored if causal estimations were affected by directionality.

A *t* test was used to compare experienced contingencies with the programmed value (Δ*p*=0). In participants who exhibited an extremely biased strategy by checking only records of patients treated with Batatrim (ie, a sampling strategy index equal to 1), no trials without the cause were sampled, and the probability of the effect in the absence of the cause *p*(*E*|~*C*)=0, and consequently experienced contingency, could not be computed since *c*/(*c* + *d*)=0/0.

To explore sampling strategies, learning phase data were split into 8 blocks of 5 trials, and a sampling strategy index was calculated for each block. A repeated measures ANOVA was used to explore the effect of block (from 1 to 8) and group (cause and effect) on sampling strategy index. Posthoc analyses (28 comparisons) were performed using Bonferroni correction.

## Results

Sampling strategy indices were significantly different from 0.50 (neutral strategy) in both groups. Participants preferentially checked the medical records of patients who, in the case of the cause group, were treated with Batatrim (mean 0.54, SD 0.17; t_104_=2.18, *P*=.03, Cohen *d*=0.21), or who, in the case of the effect group, developed the syndrome (mean 0.57, SD 0.16; t_87_=4.07, *P<*.001, Cohen *d*=0.43).

The 2×2 ANOVA demonstrated that the sampling strategy index did not differ between groups (*F*_1,189_=2.30, *P*=.13, η^2^_p_=0.01), and it was not affected by the position in which buttons were presented (*F*_1,189_=1.31, *P*=.26, η^2^_p_=0.01). The group × button position interaction also was not significant (*F*_1,189_=0.14, *P*=.71, η^2^_p_=0); therefore, both groups showed a similar sampling strategy, selecting the medical records of patients who were exposed to the potential cause or who suffered the effect more often than the medical records of patients who were not exposed to the potential cause or who did not suffer the effect.

**Figure 4:**
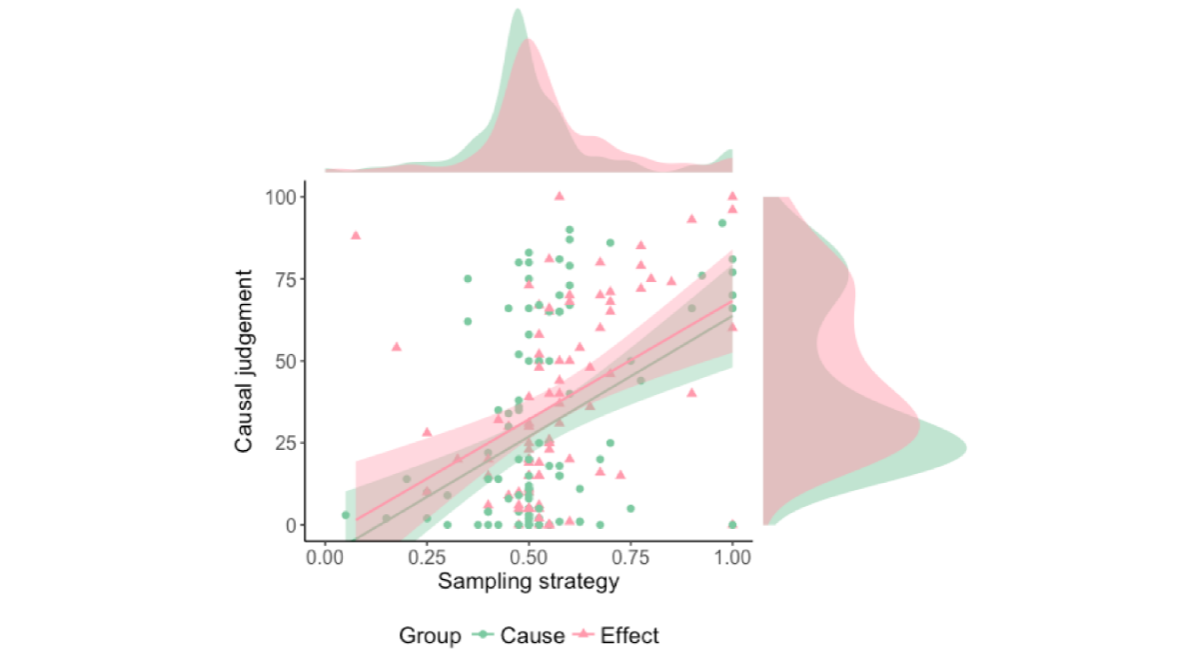
Causal estimations as a function of sampling strategy index and group.

Only a significant effect of sampling strategy index (*F*_1,185_=32.53, *P*<.001, η^2^_p_=.15) was demonstrated by the ANCOVA, suggesting that the relationship between information searching strategy and causal estimation was independent of group and directionality (Table 1 and Figure 4).

**Table 1:** Summary of ANCOVA analysis for variables predicting causal estimations.

Effect	*F* test (*df*_1_,*df*_2_)	*P* value	Partial eta square
Sampling strategy index	32.53 (1,185)	<.001	0.15
Directionality	0.61 (1,185)	.44	0
Group	0.20 (1,185)	.66	0
Sampling strategy index × directionality	0.22 (1,185)	.64	0
Sampling strategy index × group	0.01 (1,185)	.93	0
Directionality × group	1.63 (1,185)	.20	0.01
Sampling strategy index × directionality × group	1.60 (1,185)	.21	0.01

Note that any analysis that included experienced contingency did not take into account 5 participants who exhibited an extremely biased strategy (sampling strategy index=1).

No differences were found between experienced contingency and the programmed value (*p*=0) either in the cause group (t_99_=1.49, *P*=.14, Cohen *d*=0.15) or in the effect group (t_87_=0.54, *P*=.59, Cohen *d*=0.06) meaning that most participants experienced a near zero contingency. Once the 5 participants for whom it was not possible to calculate Δ*p* were discarded, no relationship between sampling strategy and experienced contingency was detected (*r*=0.06, *P*=.41); therefore, the effect of sampling strategy on causal estimation could not be attributed to experienced contingency.

**Figure 5:**
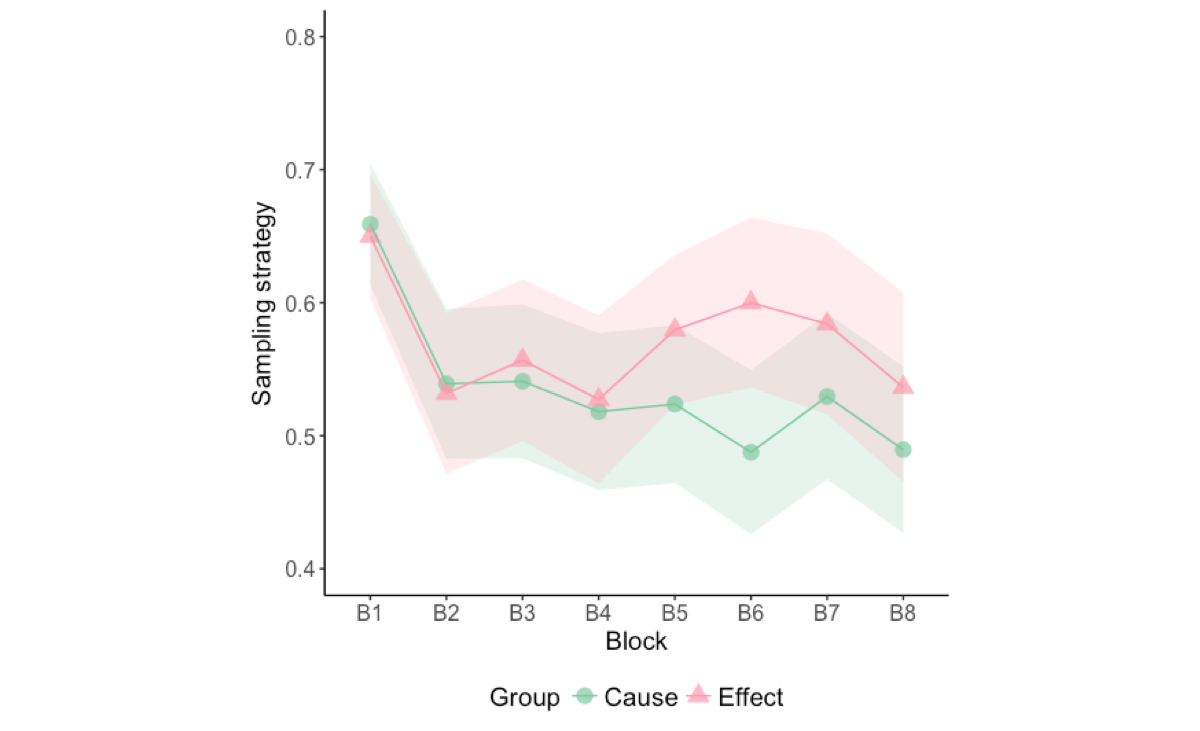
Mean sampling strategy index for each block of 5 trials for the cause group and for the effect group. Ribbons depict 95% CI.

The repeated measures ANOVA showed a significant effect of block (*F*_7,1337_=5.24, *P<*.001, η^2^_p_ =.027). The sampling strategy index was significantly higher in block 1 than in the other seven blocks, while no other significant differences were found (Table 2 and Figure 5). In block 1, 93% (98/105) of participants in the cause group selected the medical record of a patient treated with Batatrim as their first choice, and similarly, 86% (76/88) of participants in the effect group selected the medical record of a patient who developed the syndrome.

**Table 2:** Posthoc comparisons.

Comparison	Mean difference	*t* test (*df*)	*P* value (Bonferroni^a^)	*P* value (uncorrected^b^)
Block 1 - block 2	0.12	4.43 (1337)	<.001	<.001
Block 1 - block 3	0.11	3.93 (1337)	.003	<.001
Block 1 - block 4	0.13	4.90 (1337)	<.001	<.001
Block 1 - block 5	0.10	3.82 (1337)	.004	<.001
Block 1 - block 6	0.11	4.12 (1337)	.001	<.001
Block 1 - block 7	0.10	3.63 (1337)	.008	<.001
Block 1 - block 8	0.14	5.27 (1337)	<.001	<.001
Block 2 - block 3	–0.01	–0.50 (1337)	>.999	.62
Block 2 - block 4	0.01	0.47 (1337)	>.999	.64
Block 2 - block 5	–0.02	–0.60 (1337)	>.999	.55
Block 2 - block 6	–0.01	–0.31 (1337)	>.999	.76
Block 2 - block 7	–0.02	–0.79 (1337)	>.999	.43
Block 2 - block 8	0.02	0.84 (1337)	>.999	.40
Block 3 - block 4	0.03	0.97 (1337)	>.999	.33
Block 3 - block 5	–0.00	–0.10 (1337)	>.999	.92
Block 3 - block 6	0.01	0.19 (1337)	>.999	.85
Block 3 - block 7	–0.01	–0.29 (1337)	>.999	.77
Block 3 - block 8	0.04	1.34 (1337)	>.999	.18
Block 4 - block 5	–0.03	–1.08 (1337)	>.999	.28
Block 4 - block 6	–0.02	–0.79 (1337)	>.999	.43
Block 4 - block 7	–0.03	–1.27 (1337)	>.999	.21
Block 4 - block 8	0.01	0.36 (1337)	>.999	.72
Block 5 - block 6	0.01	0.29 (1337)	>.999	.77
Block 5 - block 7	–0.01	–0.19 (1337)	>.999	.85
Block 5 - block 8	0.04	1.44 (1337)	>.999	.15
Block 6 - block 7	–0.01	–0.48 (1337)	>.999	.63
Block 6 - block 8	0.03	1.15 (1337)	>.999	.25
Block 7 - block 8	0.04	1.63 (1337)	>.999	.10

^a^Bonferroni corrected values; statistically significant when *P*<.05.

^b^Uncorrected values; statistically significant when *P*<.002.

## Discussion

### Principal Results

The main goal of this experiment was to assess the potential relationship between information gathering biases and causal inferences using an experimental procedure. Thus, we adapted a standard laboratory task which has previously been used in research on causal illusions in order to imitate an information searching situation. Results showed a significant a relationship between causal illusion and information sampling strategy. When the potential cause was used to collect information, the causal link can be overestimated when cause-absent information is undersampled. Similarly, when the effect is the cue that drives information gathering, causal estimations can be overestimated when effect-absent information is insufficiently sampled.

Although we did not explicitly include any manipulation aimed at biasing sampling strategy, we found a general preference for checking the medical records of patients treated with Batatrim or patients who developed the syndrome (depending on the group). We may explain this preference as the result of a positive testing strategy driven by a confirmation bias [55]. Instructions presented the treatment with Batatrim as a potential cause for the allergic reaction: “You suspect that Lindsay syndrome may be caused by a medical treatment called Batatrim...” Consequently, we provided participants with the initial hypothesis that Batatrim caused Lindsay syndrome. People using a positive testing strategy will search for information that confirms their hypothesis. Under the initial hypothesis that Batatrim causes Lindsay syndrome, a positive testing strategy involved searching information to obtain coinciding events. When a search is based on the cause, the strategy that allows for retrieving cause-effect coincidences is to select cases in which the cause is present (ie, medical records of patients treated with Batatrim) whereas the way to obtain these coincidences when searching is driven by the effect is by selecting cases in which the effect occurred (ie, medical records of patients who developed the syndrome). These two biases resemble the sampling strategy bias detected in our experiment. Our results about the relationship between information searching strategy and causal estimation (a significant effect of sampling strategy index on causal estimation) also showed the danger associated with this testing strategy—information collected using a positive testing strategy will led to an overrepresentation of cue-present trials and will increase the likelihood of a causal illusion.

Since a positive testing strategy has been claimed to be a general default heuristic that is often used in the absence of specific information identifying some tests as more relevant than others [55], it is not necessary to assume that a confirmation bias support our results; however, previous beliefs should be taken into account when the information sampling strategies are tested in real contexts given that personal interest and motivation may exert a heavy influence boosting the effect of a default-biased strategy. The role of confirmation bias has already been explored in health information sampling research suggesting that it may significantly affect how information is collected. In recent research, Meppelink et al [56] investigated the role of confirmation bias in information seeking with respect to early-childhood vaccination and found that a priori vaccination beliefs biased selection of online health information—people predominantly selected information that was consistent with their existing beliefs (ie, selective exposure) [57]. The significant effect of sampling strategy index on causal estimations showed that, in addition, a partial selection of information in which belief-supporting evidence is overrepresented may be related to damaged causal estimation (note that the correlational nature of our design does not allow us to discriminate whether the biased searching caused the estimates to be biased, or whether a stronger initial belief about the causal relationship might have biased the sampling strategy, strengthening the initial belief).

### Limitations and Strengths

In order to ensure strong experimental control of the variables involved and a high internal validity, we decided to use a standard and very simple procedure that is often used to study how people make causal inferences in laboratory settings. Our use of this procedure in the current situation, however, resulted in limitations related to its ecological validity. Our procedure does not exactly mirror how internet users search for information. Most internet users do not sequentially select information about individual people in the same way as was done in the experimental task; however, the process of collecting information and the subsequent processes of integration, combination, and interpretation of the information are, fundamentally, the same.

Participants searched for and collected pieces of evidence that ultimately were used to shape their estimation about the relationship between the events. Similarly, internet users may use web search engines which provide them with discrete bits of information that are used as evidence to support or reject the causal relationship between the events under assessment. In our experiment, these pieces of information were less enriched than those collected in real-world settings, but they did contain the core information needed for causal inference. Reducing the ecological validity of our procedure ensured a high level of internal validity—an advantage that made our procedure a better option than other naturalistic paradigms. The most relevant advantage was that it allowed us to explore information sampling biases while controlling for the effect of additional information features.

Real situations contain a high degree of ambiguity and subtle information nuances that may limit research inferences by weakening internal validity. For example, website design or the perceived authority of the author have been shown to influence the trust and credibility of web-based health information [58], which may affect how specific pieces of information are weighted and integrated to make causal inferences. Instead, the experimental approach allowed for the isolation of searching strategy from other factors. Another advantage of the experimental approach was that we were able to control which information was presented, and consequently, whether the information objectively supported any relationship between the cause and the alleged effect in order to detect causal illusions. Finally, by using a fictitious scenario instead of a real-world example, we avoided the potential consequences of experimentally induced causal beliefs on real-life decisions and controlled for any contribution of a priori beliefs.

### Future Work

Now that the contribution of sampling strategies on causal inferences has been documented in a laboratory setting, future research may extend our results to real-world situations to assess the generalizability of our findings when information collection is more complex. This research may be considered a first step in building interventions aimed at protecting people when using the internet to search health information.

### Conclusions

The internet has become a relevant source of health-related information [6-8]. Despite its advantages, using the internet to gather information requires several considerations such as the lack of quality control of the information and the subsequent possibility of misinformation dissemination. A relevant example is misinformation concerning scientific strategies that are aimed at protecting and promoting public health such as vaccination. Although, the determinants of vaccine uptake are complex, online misinformation has been claimed to contribute to the phenomenon of individuals foregoing vaccinations [59,60] and major search engines and social media organizations have been recently called to actively support fact-based communication programs that positively contribute to restoring confidence in vaccinations [61]. Using the internet to gather health information may cause additional concerns beyond those of information quality. How people search for information may determine which information is retrieved [62,63], shaping their beliefs about health, and eventually, their health-related behavior, such as vaccination refusal [64,65]. Results from our study have shown that sampling biases are related to causal perceptions. Thus, partial selection of information may induce an uneven representation of information that may produce and perpetuate causal illusions.

Laboratory-based research on contingency learning has been shown to be a successful approach to real-life problems because of its ability to detect relevant factors that may contribute to causal inferences, but also because it has been the foundation for designing and testing evidence-based interventions that have proven to be effective in improving critical thinking skills, and therefore, at reducing potentially harmful causal misconceptions in real contexts [48,49]. Future research may extend our results to real-world contexts in order to design interventions aimed at protecting users when using the internet.

## Appendix

Multimedia Appendix 1 Detailed instructions used in the allergy task.

## Abbreviations

ANCOVA: analysis of covariance
ANOVA: analysis of variance
