# Supplementary material for: Biased Sampling and Causal Estimation of Health-Related Information: Laboratory-Based Experimental Research
Source: J Med Internet Res. 2020 Jul 24;22(7):e17502. doi: 10.2196/17502 (PMC7414405; doi:10.2196/17502)
Supplement: Multimedia Appendix 1 [file jmir_v22i7e17502_app1.docx]

## Multimedia Appendix 1

Detailed instructions used in the Allergy task

**[Instructions Screen 1]**

**All groups:** Imagine that you are a doctor who works in a hospital. Some of your patients are developing Lindsay's Syndrome while they are hospitalized. This syndrome is a severe allergic reaction characterized by skin alterations and transient hair loss:


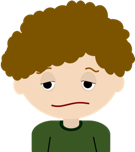


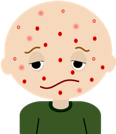


*Patient with Lindsay's Syndrome (left) and without Lindsay's Syndrome (right)*

You suspect that Lindsay's Syndrome may be caused by a medical treatment called Batatrim that is sometimes used in the hospital, so you have requested the hospital for an authorization to check the treatments of previous patients.

**[Instructions Screen 2]**

**Group Cause:** The hospital has authorized your research. You will be able to check the medical records of previous patients, but you will have to apply for them one by one. On each application you will have to indicate if you want to check the medical records of a patient treated with Batatrim or the records of a patient that was not treated with Batatrim. The hospital will then let you know if the patient developed Lindsay's Syndrome or not.

Your mission is to discover whether the treatment with Batatrim causes Lindsay's Syndrome.

**Group Effect:** The hospital has authorized your research. You will be able to check the medical records of previous patients, but you will have to apply for them one by one. On each application you will have to indicate if you want to check the medical records of a patient who developed Lindsay's Syndrome or the records of a patient that did not develop Lindsay's Syndrome. The hospital will then let you know if the patient was treated with Batatrim or not.

Your mission is to discover whether Lindsay's Syndrome is caused by the treatment with Batatrim.
